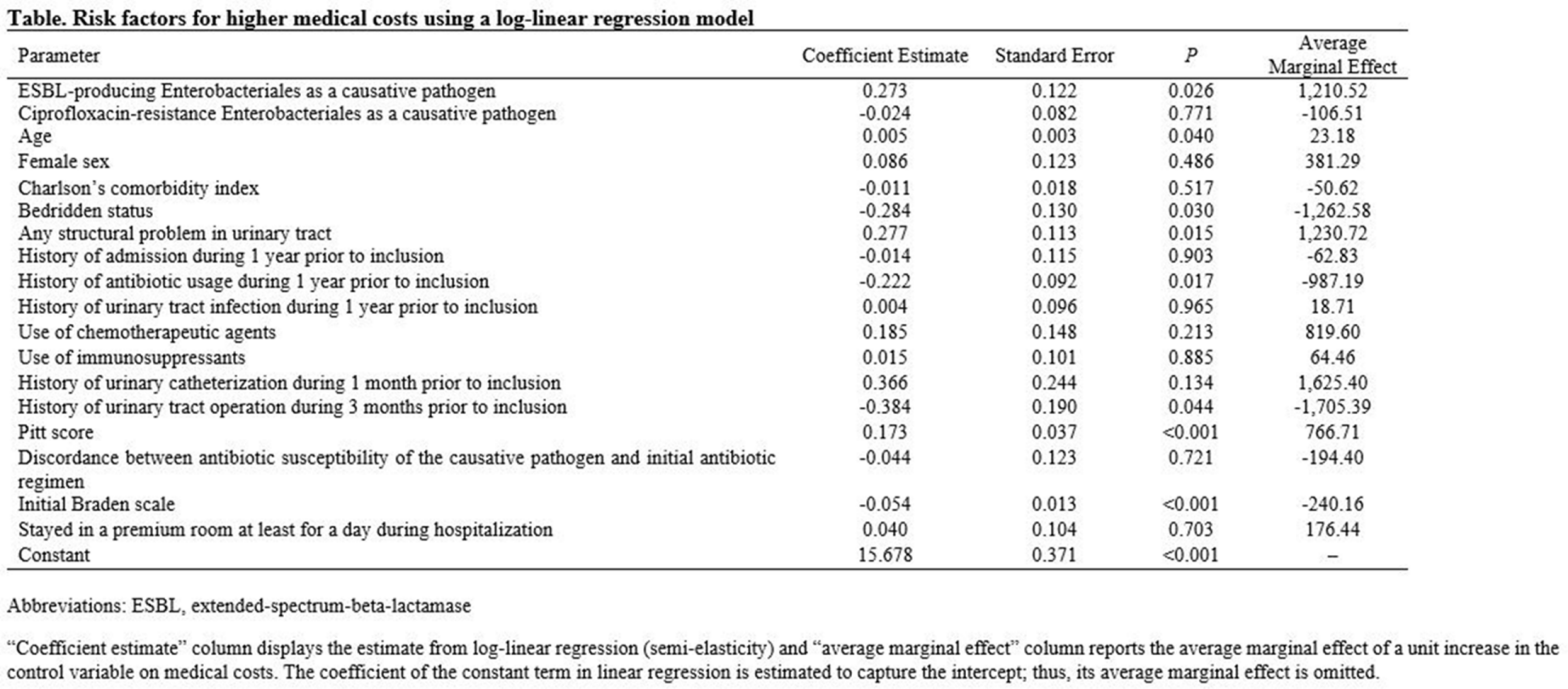# How Does Antimicrobial Resistance Increase Medical Costs in Community-Acquired Acute Pyelonephritis?

**DOI:** 10.1017/ash.2021.42

**Published:** 2021-07-29

**Authors:** Bongyoung Kim, Taul Cheong, Jungmo Ahn

## Abstract

**Background:** The proportion of antimicrobial-resistant Enterobacterales that are causative pathogens for community-acquired acute pyelonephritis (CA-APN) has been increasing. We examined the effect of antimicrobial resistance on medical costs in CA-APN. **Methods:** A single-center retrospective cohort study was conducted at a tertiary-care hospital in Korea between January 2018 to December 2019. All hospitalized patients aged ≥19 years who were diagnosed with CA-APN were recruited, and those with Enterobacterales as a causative pathogen were included. Comparisons between CA-APN caused by extended-spectrum β-lactamase (ESBL)–producing pathogens (ESBL+ group) and those by non–ESBL-producing organisms (ESBL– group) as well as CA-APN caused by ciprofloxacin-resistant pathogens (CIP-R group) and those by ciprofloxacin-sensitive pathogens (CIP-S group) were performed. Log-linear regression was performed to determine the risk factors for medical costs. **Results**: In total, 241 patients were included in this study. Of these, 75 (31.1%) had an ESBL-producing pathogen and 87 (36.1%) had a ciprofloxacin-resistant pathogen. The overall medical costs were significantly higher in the ESBL+ group compared with the ESBL− group (US$3,730.18 vs US$3,119.32) P <0.001) as well as in CIP-R group compared with CIP-S group (3,730.18 USD vs. 3,119.32 USD, P =0.005). In addition, length of stay was longer in ESBL+ group compared with ESBL-group (11 vs. 8 days, P <0.001) as well as in CIP-R group compared with CIP-S group (11 vs. 8 days, P <0.001). There were no significant difference in the proportion of clinical failure between ESBL+ and ESBL- groups; CIP-R and CIP-S groups. Based on the log-linear regression model, the costs associated with ESBL-producing Enterobacterales as the causative pathogen would be, on average, 27% higher or US$1,211 higher than its counterpart (*P* = .026). By the same token, a patient who is a year older would incur US$23 higher cost (*P* = .040). Having any structural problem in urinary tract would incur US$1,231 higher cost (*P* = .015). A unit increase in Pitt score would incur US$767 USD higher cost (P < 0.001) higher cost, all other things constant. **Conclusions**: Medical costs for hospitalized patients with CA-APN are increased by the existence of ESBL-producing Enterobacterales but not by the existence of ciprofloxacin-resistant Enterobacterales.

**Funding:** No

**Disclosures:** None

**Figure 1.**
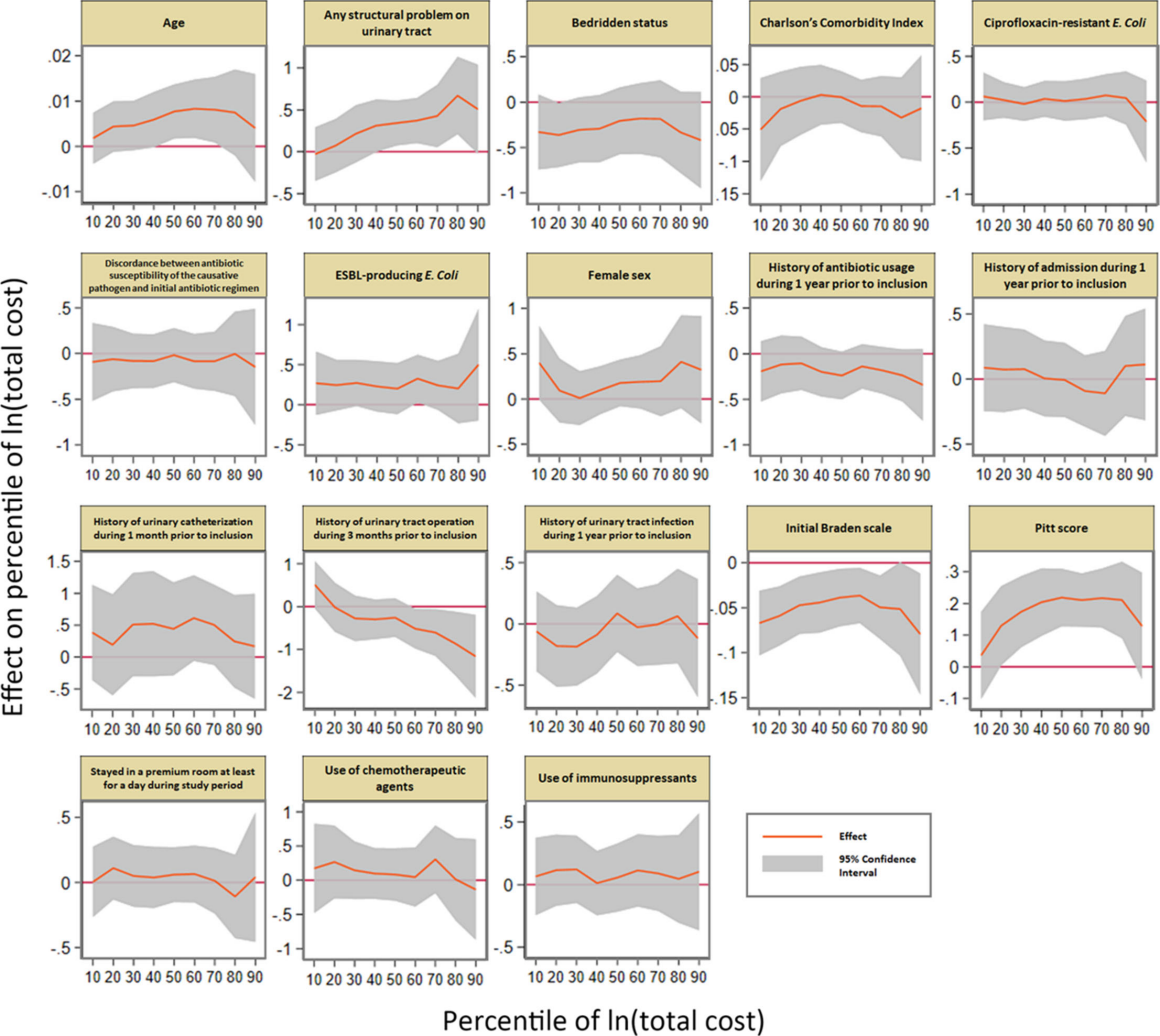

**Table 1.**
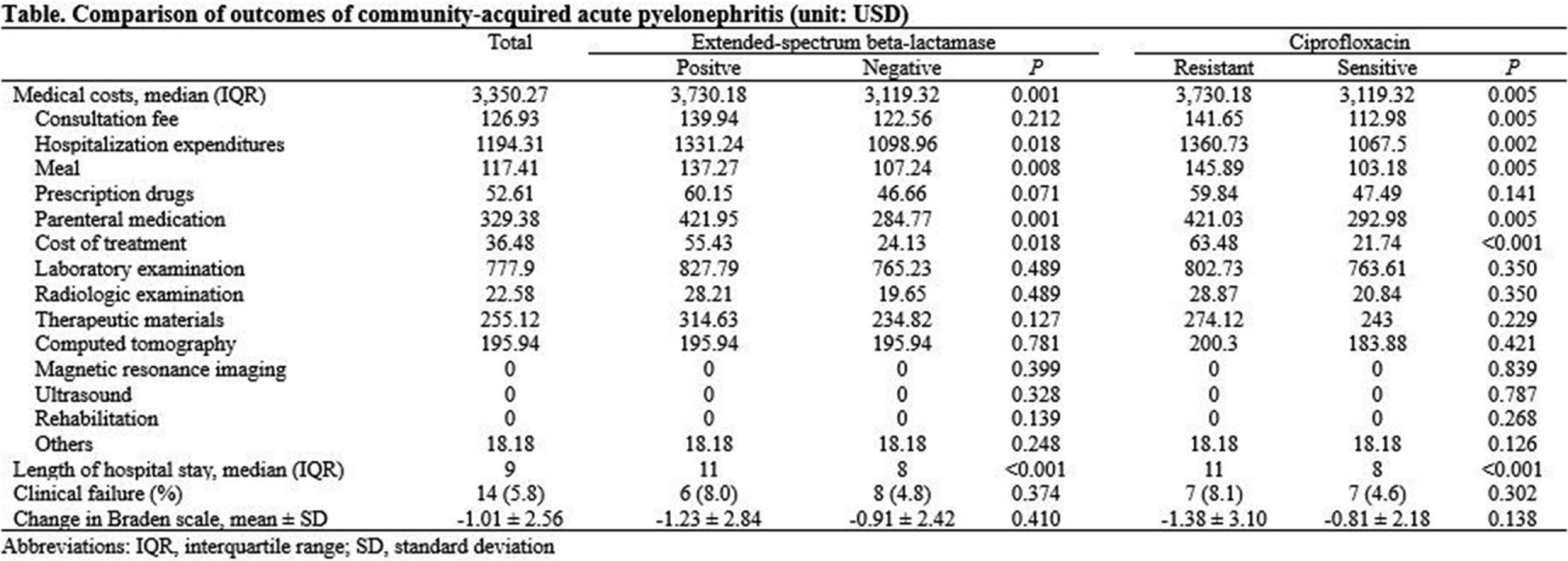

**Table 2.**